# Gate-Controlled WSe_2_ Transistors Using a Buried Triple-Gate Structure

**DOI:** 10.1186/s11671-016-1728-7

**Published:** 2016-11-22

**Authors:** M. R. Müller, R. Salazar, S. Fathipour, H. Xu, K. Kallis, U. Künzelmann, A. Seabaugh, J. Appenzeller, J. Knoch

**Affiliations:** 1 Intelligent Microsystems Chair, TU Dortmund, Emil-Figge-Str. 68, 44227 Dortmund, Germany; 2 Birck Nanotechnology Center, Purdue University, 1205 W. State Street, West Lafayette, IN 47907 USA; 3 Department of Electrical Engineering, University of Notre Dame, Notre Dame, IN 46556 USA; 4 Institute of Semiconductor and Microsystems Technology, TU Dresden, 01062 Dresden, Germany; 5 Institute of Semiconductor Electronics, RWTH Aachen University, Sommerfeldstr. 24, 52074 Aachen, Germany; 6 Present address: Infineon Technologies, Siemensstrasse 2, 9500 Villach, Austria

**Keywords:** Electrostatic doping, Tungsten diselenide (WSe_2_), Reconfigurable device

## Abstract

In the present paper, we show tungsten diselenide (WSe_2_) devices that can be tuned to operate as *n*-type and *p*-type field-effect transistors (FETs) as well as band-to-band tunnel transistors on the same flake. Source, channel, and drain areas of the WSe_2_ flake are adjusted, using buried triple-gate substrates with three independently controllable gates. The device characteristics found in the tunnel transistor configuration are determined by the particular geometry of the buried triple-gate structure, consistent with a simple estimation of the expected off-state behavior.

## Background

In recent years, two-dimensional layered materials, such as graphene and transition metal dichalcogenides (TMDs), have attracted a great deal of interest as channel material in high-performance field-effect transistors (FETs) [1–8]. A major advantage of these materials is their ability to drive high electrical currents in only few layers or even a monolayer (<1 nm). In addition, there are a number of semiconducting TMDs that exhibit band gaps ≥ 1 eV. Thus, they are in principle ideally suited for ultimately scaled FETs as well as for band-to-band tunnel FETs tunnel field-effect transistors (TFETs) since the ultrathin channel layer enables excellent gate control yielding ultimate scalability and a large band-to-band tunnel probability [9–16].

However, a major challenge for the application of TMDs is the realization of an appropriate source-channel-drain doping profile because conventional (e.g., impurity, chemical) doping is extremely challenging [17, 18], and in the case of TFETs, the right amount of doping is subject to tedious optimization: if the doping concentration is too small, the gate action is not sufficiently screened, and the band-to-band tunneling probability cannot be made large enough. On the other hand, a large doping concentration may lead to a large Fermi energy, and as a result, carriers are injected from the Boltzmann tail of the source Fermi distribution function giving rise to a TFET with an inverse subthreshold slope close to 60 mV/dec [19–21]. A viable alternative is source-drain doping by means of electrostatic potentials, which has already been employed to investigate carbon nanotube, graphene, and nanowire FETs as well as optoelectronic devices [9, 22–28]. This approach not only allows convenient doping, but in a TFET, it enables to disentangle screening in source from the position of the Fermi energy [21]. Moreover, electrostatic doping adds device level functionality through the tunability to operate the same device in various transistor configurations. Specifically, if the electrostatic doping is controllable individually for source and drain, not only the *n*-type and *p*-type FETs can be realized [29] but the device can also be switched into a TFET mode of operation which allows tuning to devices from conventional to low-power operation.

Here, we present a tungsten diselenide (WSe_2_) device which we tune by electrostatic doping to function as an *n*FET (*n*-*n* doping profile), *p*FET (*p*-*p* doping profile), and TFET (*n*-*p* doping profile). WSe_2_ is used here since, in contrast to molybdenum disulfide (MoS_2_), it provides an approximately mid-gape Fermi level line-up for (e.g., nickel) ensuring that electrons as well as holes can be injected efficiently in the different device configurations [30]. In addition, it exhibits a smaller band gap and lower effective masses compared to MoS_2_, both known to improve the performance of TFETs [13–16, 21]. The electrostatic doping is realized by a modified version of our previously employed buried triple-gate (BTG) structure [31]. This structure features three independently controllable gates: one center-gate and two side-gates for electrostatic doping. Since all three gates are buried and the BTG’s surface is entirely flat, the structure serves as a platform for convenient deposition and investigation of devices from various kinds of novel channel materials. The electrostatic environment is fully determined by the BTG structure, therefore, allows true comparison of various materials in terms of their suitability for future FET or TFET devices.

## Methods

**Fig. 1:**
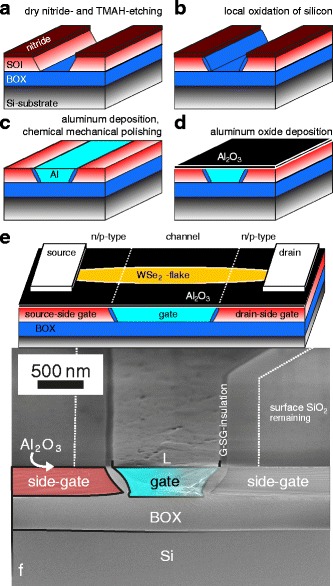
**a**–**d** Manufacturing process of the LOCOS BTG structure. **e** Schematic cross-section and bird’s-eye view of a WSe_2_ device. **f** Scanning electron microscopy cross-section and surface micrograph (at an angle of 80° from the top view) of the LOCOS BTG structure

Figure 1 illustrates the manufacturing process of the BTG structure. The starting material is a 4′′ silicon-on-insulator (SOI) wafer with a top layer of 340 nm (100) Si and a 400 nm buried oxide (BOX). The silicon is implanted with 10^15^ cm^−2^ of phosphorous at 75 keV, 7.5°, yielding a peak implantation depth of 104 nm. Afterwards, the wafer is RCA-cleaned, and a 200-nm layer of Si_3_N_4_ is deposited by a plasma-enhanced chemical vapor deposition. Subsequently, a furnace treatment at 900 °C for 20 min heals the implantation damage, activates the dopants, and condenses the nitride. Optical lithography in combination with CHF_3_ + O_2_ plasma etching is used to pattern the nitride layer. After the removal of the photoresist (PR) and directly after an additional buffered oxide etch dip, tetramethylammonium hydroxide (TMAH), 25 wt% at 80 °C, is used to anisotropically etch through the SOI top layer (cf. Fig. 1a). Wet thermal oxidation is used to grow the gate-side-gate (G-SG) insulation (Fig. 1b). Afterwards, the nitride mask is removed by a two-step procedure: (i) CHF_3_ + O_2_ plasma removes the oxidized nitride and (ii) H_3_PO_4_ at 160 °C removes the remaining nitride. No apparent oxidation under the nitride mask (bird’s beak) is present. A thin oxide (~10 nm) is grown on the silicon surface in order to serve as an additional insulation and as a stopping layer for the following damascene process: After sputter-deposition of aluminum onto the surface, chemical–mechanical planarization (CMP) is employed to remove the Al overburden yielding an entirely planar structure (Fig. 1c). To reduce the thermally induced growth of hillocks on the Al-surface, a repolishing procedure is used [32]. The gate dielectric is obtained by atomic layer deposition (ALD) of 7 nm Al_2_O_3_ (Fig. 1d). The BTG structures are completed by selectively etching the oxides using CHF_3_ + Ar plasma in the contact regions and subsequent deposition of 10 nm Ti + 170 nm Au as side and top-gate contact metals using a lift-off technique. The finished wafer is cut into sample pieces of 7.5 × 7.5 mm^2^; Fig. 1e shows a schematic device cross-section and bird’s-eye view.

Figure 1f presents a scanning electron microscopy (SEM) micrograph of the BTG’s surface and cross-section. Clearly visible is the G-SG insulation of 90 nm. Not visible is the 7 nm Al_2_O_3_ dielectric cap layer on the surface. An excellent topography, with a dishing of only ~1 nm per micron, and quality (only few pits and scratches, roughness down to 1-nm root mean square) of the polished aluminum is achieved [32, 33].

**Fig. 2:**
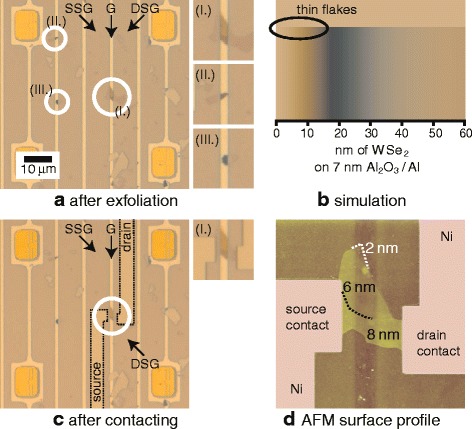
Fabrication process of WSe_2_ devices on the LOCOS BTG structure: **a** Exfoliation of WSe_2_ flakes on the structure, **b** visibility simulation, **c** fabrication of source/drain contacts, and **d** AFM surface profile to determine the thickness of the flake

For the fabrication of the WSe_2_ devices on the BTG structure, first, WSe_2_ is exfoliated from bulk material using a blue tack tape. Figure 2a shows a section of a BTG meander with additional electron-beam lithography markers. The sample is inspected using optical microscopy in order to identify thin flakes (<10 nm) lying across the aluminum gate, e.g., a flake as in location (I.). Thin flakes were found to exhibit a characteristic yellowish color on the gate region (Al_2_O_3_/Al) with a strong contrast to thicker flakes of ~15–25 nm, which appear distinctly blue-colored (cf. locations (II.) and (III.)). Interestingly, such a clear distinction is not present on the side-gate region (Al_2_O_3_/SiO_2_/Si/SiO_2_/Si). The characteristic swing from blue to yellowish colors identifying the thinnest flakes is also confirmed by our simulations, as shown in Fig. 2b [34].

After identification of appropriate flakes, electron-beam lithography is used to pattern source/drain contact structures into polymethyl methacrylate (PMMA). Subsequently, deposition of 90 nm nickel and lift-off are employed to obtain the contact structures and complete the devices. Figure 2c depicts the flake from (a) after contact formation. In order to determine the thickness of the flakes, atomic force microscopy (AFM) is conducted. Figure 2d shows the AFM surface profile of the presented flake, indicating a thickness of ~6–8 nm.

## Results and Discussion

**Fig. 3:**
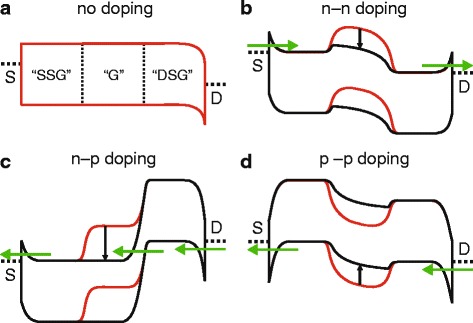
Schematic band diagrams for electrostatically doped *n*FET, *p*FET, and TFET devices. **a** Illustration in the case of zero gate voltage in source, drain, and gate area. **b** Positive side-gate voltages create *n*-type regions in source and drain, **c** a positive side-gate voltage in source and a negative side-gate voltage in drain yield a tunnel FET device. **d** In the case of negative side-gate voltages, *p*-type source/drain electrodes are realized

**Fig. 4:**
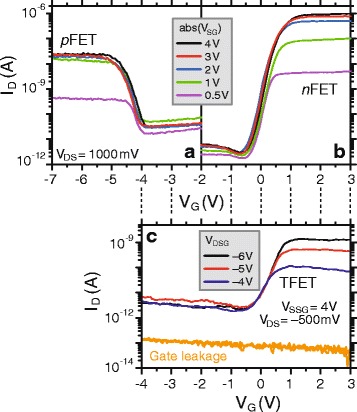
Transfer characteristics of a ~3-nm-thick WSe_2_ flake, electrostatically doped to function as **a** an *n*FET (*V*_SG_ > 0) and **b** *p*FET (*V*_SG_ < 0). Note that the gate leakage current is always less than 10^−12^A; for clarity, it is not shown in **a** and **b**. **c** Device in TFET configuration

Figure 3 illustrates the operational principle of our devices: For the realization of an *n*FET (Fig. 3b), positive and equal voltage is applied to both side-gates (this means that *V*_SSG_ = *V*_DSG_ > 0. For simplicity, this will be called side-gate voltage *V*_SG_ in the following) which lowers the bands in the regions controlled by the side-gates (SSG and DSG) and yields an *n*-*n* doping profile. Note that all given voltages are in reference to the ground potential at the source contact. The described electrostatic doping yields a thinning of the source and drain Schottky barriers (SB) for electron injection while suppressing hole injection at the same time. As a result, a unipolar device behavior is expected in contrast to the usually observed ambipolar behavior of WSe_2_ transistors. Applying positive voltage to the center gate, i.e., the actual gate of the device (denoted with *V*_G_ in the following), yields a lowering of the bands in the gate-controlled region (G) and switches the device on. The described mechanism can be employed similarly to realize a *p*FET device (i.e., a *p*-*p* doping profile) by applying *V*_SG_ < 0, *V*_G_ < 0 (cf. Fig. 3d). Figure 4 displays transfer characteristics of a WSe_2_ with a thickness of *d*_WSe2_ = 3 nm determined by AFM. The source-drain bias (*V*_DS_) is constant at 1 V and the various curves presented are from different side-gate voltages. The *n*-doping (Fig. 4a) leads to an increase of the drain current up to an *I*_on_/*I*_off_ ratio of >3 × 10^5^ at *V*_SG_ = 4 V. As already mentioned above, a *p*FET device is realized for *V*_SG_ < 0, *V*_G_ < 0. In this case, we find an *I*_on_/*I*_off_ ratio of ~10^3^ at *V*_SG_ = −4 V (cf. Fig. 4b). The ~100× larger *n*-current in comparison to the *p*-current (as well as the larger leakage current on the *p*FET compared to the *n*FET) is attributed to the influence of the nickel contacts, which were shown to be slightly more *n*-type [7, 30]. The subthreshold swings are 180 mV/dec (*n*FET) and 240 mV/dec (*p*FET), respectively. Note, that the current for large positive (*n*-type device) and negative (*p*-type device) gate voltages *V*_G_ saturates, and current flow is then limited by the injection through the source-side SB. Since the tunneling probability is a function of the barrier height (0.54 and 0.74 eV in the case of electron and hole injection, respectively) and effective mass (0.34 and 0.44 m_0_ for electrons and holes, respectively), a different modulation of the tunneling probability through the SB is obtained for the *n*-type and the *p*-type [30]. As a result, the side-gates have considerably less impact on the SB in the *p*-type device which is reflected in the drain current for *abs*(*V*_SG_) *> 2 V* that hardly changes anymore.

In addition, for this device we are also able to set up a TFET device configuration with an *n*-type source and a *p*-type drain contact using *V*_DS_ = −500 mV, *V*_SSG_ = 4 V, *V*_DSG_ <−4 V. As displayed in Fig. 4c, the gate leakage current in the TFET device configuration is at least 30× lower than the drain current. It is important to note that usually temperature-dependent measurements of the off-state are used to demonstrate TFET behavior. However, in the present case, we were able to demonstrate *n*- and *p*-type device behavior proving that a conduction-/valence-band profile appropriate for TFETs can be adjusted using the BTG. And since gate leakage can be neglected, we ascribe the exponential increase of current around *V*_G_ = 0 V to band-to-band tunneling. This is also consistent with the fact that increasingly negative DSG voltages improve the band-to-band tunneling probability (by increasing the electric field at the G/DSG junction) while at the same time hinder thermionic transport due to an increased potential barrier carriers from drain have to overcome.

The *I*_on_/*I*_off_ ratio is ~4.5 × 10^2^ at *V*_DSG_ = −6 V, and the inverse subthreshold slope is *S ≈* 366 mV/dec. This rather large value is due to the fact that overpolishing during the CMP fabrication process leads to a G-SG insulation of approximately *d*_G-SG_ = 90 nm (cf. Fig. 1f). Interpreting the band-to-band tunneling barrier as a SB, the expected inverse subthreshold slope can be computed with $S\approx {k}_BT/q\cdot ln(10)\;\left(1/2+1/3\cdot \sqrt{d_{\mathrm{G}\hbox{-} \mathrm{S}\mathrm{G}}{d}_{\mathrm{WSe}2}}\right)$ [35, 36] yielding *S* ≈ 358 mV/dec. This agrees very well with the experimental value showing that it is the G-SG insulation that limits the performance of the device with *n*-*p* doping. Further improvements in the BTG fabrication will therefore allow a substantial performance increase. In addition, the channel layer thickness, i.e., the WSe_2_ flake thickness, plays a prominent role for the device performance of the TFETs [15, 16]. Indeed, in the case of a device with an approximately 13-nm-thick WSe_2_ flake, we found a strongly deteriorated TFET behavior (not shown here). In turn, a thinner flake should allow substantial improvements in terms of inverse subthreshold slope and on-state current in the TFET configuration.

From the mere consideration of the band gap of WSe_2_, we would expect a larger on/off current ratio with significantly smaller off-state currents. However, an explanation of this phenomenon requires a detailed simulation of the device structure including the fact that in the present device, the distribution of the injected current among the various WSe_2_ layers [37] depends on the different (side-) gate voltages. We therefore speculate that band-to-band tunneling as well as interlayer coupling is responsible for the electrical behavior of the device in TFET configuration.

## Conclusions

We demonstrated that electrostatic doping allows the realization of a tunable device to function as *n*FET, *p*FET, and as band-to-band tunnel FET on the same WSe_2_ flake. Based on a BTG substrate, we were able to dope source and drain regions independently to create the required band-profiles for the three transistor configurations. An optimized triple-gate configuration thus enables to dynamically switch transistors from conventional (*n*FET, *p*FET) to low-power (TFET) operation.
